# Altered volume of the amygdala subregions in patients with chronic low back pain

**DOI:** 10.3389/fneur.2024.1351335

**Published:** 2024-03-28

**Authors:** Si-Yu Gu, Feng-Chao Shi, Shu Wang, Cheng-Yu Wang, Xin-Xin Yao, Yi-Fan Sun, Jian-Bin Hu, Fei Chen, Ping-Lei Pan, Wen-Hui Li

**Affiliations:** ^1^Department of Radiology, Affiliated Hospital 6 of Nantong University, Yancheng Third People’s Hospital, Yancheng, China; ^2^Department of Orthopedics, Affiliated Hospital 6 of Nantong University, Yancheng Third People’s Hospital, Yancheng, China; ^3^Department of Central Laboratory, Affiliated Hospital 6 of Nantong University, Yancheng Third People’s Hospital, Yancheng, China

**Keywords:** chronic low back pain, amygdala subregions, FreeSurfer, magnetic resonance imaging, structural neuroimaging

## Abstract

**Background:**

Neuroimaging studies have suggested a pivotal role for the amygdala involvement in chronic low back pain (CLBP). However, the relationship between the amygdala subregions and CLBP has not yet been delineated. This study aimed to analyze whether the amygdala subregions were linked to the development of CLBP.

**Methods:**

A total of 45 patients with CLBP and 45 healthy controls (HCs) were included in this study. All subjects were asked to complete a three-dimensional T1-weighted magnetic resonance imaging (3D-T1 MRI) scan. FreeSurfer 7.3.2 was applied to preprocess the structural MRI images and segment the amygdala into nine subregions. Afterwards, comparisons were made between the two groups in terms of the volumes of the amygdala subregions. Correlation analysis is utilized to examine the relationship between the amygdala subregion and the scale scores, as well as the pain duration in patients with CLBP. Additionally, logistic regression was used to explore the risk of the amygdala and its subregions for CLBP.

**Results:**

In comparison to HCs, patients with CLBP exhibited a significant enlargement of the left central nucleus (Ce) and left cortical nucleus (Co). Furthermore, the increased volume of the left Ce was associated with a higher risk of CLBP.

**Conclusion:**

Our study suggests that the left Ce and left Co may be involved in the pathophysiological processes of CLBP. Moreover, the volume of the left Ce may be a biomarker for detecting the risk of CLBP.

## 1 Introduction

Chronic low back pain (CLBP) is the leading cause of disability globally (1), affecting approximately 20% of the global population (2). Despite such high prevalence and social burden, the pathophysiology of CLBP remains obscure (3). Studies in neuroimaging have revealed that individuals suffering from CLBP have structural changes in their brains that are not present in healthy people (4–10). For example, alterations in gray matter volume were found in several brain regions such as the posterior parietal cortex, anterolateral prefrontal cortex, left precuneus, bilateral putamen, and temporal lobe, etc. (4–7). Meanwhile, several brain regions showed changes in cortical thickness, like the paracentral lobule, right rostral middle frontal gyrus, and left medial temporal lobe, etc. (8–10). These findings indicate that the pathogenesis of CLBP is related to structural alterations in the brain.

The amygdala, a limbic brain region, is essential in managing pain and affects the emotional and cognitive responses to it (11, 12). Structural neuroimaging studies have demonstrated alterations in the volume of the amygdala in patients with CLBP, and these changes may be linked to their impaired cognitive-affective and emotional processing abilities, as well as to a more persistent chronic pain condition (6, 13, 14). However, these investigations have focused only on the total amygdala, not reflecting the subtle changes in the subregions of the amygdala.

In recent years, an atlas of the amygdala at the sub-regional level, which was created by combining ultra-high resolution *ex vivo* MRI with *in vivo* data, is available in FreeSurfer 6 and later versions^1^ (15). This statistical atlas divides the amygdala into nine subregions, including the anterior amygdaloid area (AAA), cortico-amygdaloid transition area (CAT), basal nucleus (Ba), lateral nucleus (La), paralaminar nucleus (PL), accessory basal nucleus (AB), medial nucleus (Me), central nucleus (Ce), and cortical nucleus (Co). AAA is populated by magnocellular cholinergic neurons that secrete acetylcholine, and it plays a role in preserving attention and memory (16, 17). CAT is likely to play an important role in social communication and may be linked to the assessment of negative emotions (18–20). PL is thought to be part of the Ba (21, 22), which is linked to emotion, cognition, and has the ability to regulate voluntary motor movements (23). La is the primary input structure to the amygdala, and it is believed to be a major factor in emotional processing and responses, as well as in stress-inducing stimuli (24, 25). AB is linked internally within the basal nucleus and receives input from the cortex and other subcortical regions (26). Me is considered to be the core of the neuroendocrine system that processes social data and is strongly associated with the control of defensive behavior (27, 28). Ce, the nociceptive center in the brain, is critical for pain regulation and processing and is the main output nucleus of the amygdala that drives pain-related functions (29, 30). Co is mainly responsible for olfactory processing, and its more posterior section is linked to memory processes (31–33). By utilizing FreeSurfer, we can accurately assess minor alterations in the amygdala subregions.

To date, no studies have been conducted to determine if there are any volumetric changes in the subregions of the amygdala in those with CLBP. Therefore, in this study, we hypothesized that the volumes of the amygdala subregions changed in patients with CLBP and that these alterations may be involved in the development of CLBP.

## 2 Methods

### 2.1 Participants

From the period of January 2021 to August 2022, a total of 45 patients with CLBP were recruited from the Sixth Affiliated Hospital of Nantong University (Yancheng Third People’s Hospital). Healthy controls (HCs) were enrolled from the Yancheng area through advertisements. The Yancheng Third People’s Hospital Ethics Committee approved this study, and all participants gave written informed consent.

The inclusion criteria for patients with CLBP were pain persisting or fluctuating for more than 3 months (34), Visual Analogue Scale (VAS) scores of three points or higher, no history of neuropsychiatric disorders, major systemic diseases, head injuries or comas, other chronic pain disorders, and right-handedness. All of the patients took analgesic medication on a regular basis, and Table 1 listed the types of analgesics they used. Meanwhile, HCs with no current experience of pain or past history of chronic pain, nervous system diseases, diabetes, hypertension, hyperlipidemia, mental illnesses, long-term smoking, or alcoholism were included. If patients with CLBP or HCs could not complete MRI scans, had organic brain lesions, or had contraindications to MRI, they would be excluded.

**Table 1:** The use of analgesics in patients with CLBP.

	CLBP
NSAIDs	21
Acetaminophen	10
Muscle relaxants	3
NSAIDs and acetaminophen	6
NSAIDs and muscle relaxants	3
Acetaminophen and muscle relaxants	2

### 2.2 Clinical assessment

VAS and the Oswestry Disability Index (ODI) were employed to measure the severity of pain and dysfunction experienced by patients with CLBP before MRI scans. Utilizing VAS, patients were asked to make a mark on a 10 cm line based on their perception of the pain intensity (35). The further along the line they marked, the more intense the pain. Meanwhile, ODI, comprising pain intensity, personal care, lifting, walking, sitting, standing, sleeping, sex life, social life, and traveling, was used to measure the extent of dysfunction in patients, with scores that increased as dysfunction became more severe (36).

### 2.3 MRI data acquisition

All participants underwent three-dimensional T1-weighted (3D-T1) structural imaging at a 3.0 Tesla scanner (Discovery 750w, GE, United States) at the Sixth Affiliated Hospital of Nantong University. Parameters of the sequence were as follows: Repetition time (TR) = 7.5 ms, Echo time (TE) = 2.8 ms, Field of View (FOV) = 24 cm × 24 cm, Slice thickness = 1.0 mm, Number of slices = 152, Flip Angle = 15°, Voxel size = 0.5 × 0.5 × 1 mm.

### 2.4 Amygdala segmentation

A standard FreeSurfer recon-all pipeline was employed for pre-processing T1-weighted MRI (version 7.3.2) (see text footnote 1). Then, automated amygdala segmentation and volume calculation was achieved through the use of the FreeSurfer 7.3.2 automated pipeline for subcortical structure segmentation (15). After segmentation was done, one researcher visually checked the segmentation results to ensure they were accurate.

### 2.5 Statistical analysis

Statistical analysis was carried out using Statistical Package for the Social Sciences version 27 (SPSS 27). Initially, we utilized the Shapiro–Wilk test to assess the normal distribution of age, clinical scale scores, pain duration, and intracranial volume (ICV) in patients with CLBP and HCs. If the normal distribution was met, we then used the Student’s *t*-test to evaluate the difference between the two groups; otherwise, the Mann–Whitney U test was applied. Meanwhile, the Chi-square test was used to compare the gender differences between the two groups. The significance level of the results was set at *p* < 0.05.

Cook’s Distance was utilized to assess possible outliers within the volume of the amygdala and its subregions. After that, the Shapiro–Wilk test was employed to determine if the volume of the amygdala and its subregions in patients with CLBP and HCs followed a normal distribution. The Student’s *t*-test or Mann–Whitney U test was then utilized to determine any differences between the two groups, and *p* < 0.05 was considered significant. Subsequently, Pearson or Spearman correlation analyses were used to evaluate the association of the volumes of the amygdala and its subregions with the clinical scale scores, as well as the pain duration in patients with CLBP.

Finally, logistic regression was employed to investigate the correlations between the amygdala and its subregions and the risk of CLBP. Odds ratios (ORs) and 95% confidence intervals (CIs) were evaluated using univariate and multivariate models. The logistic regression analysis involved the implementation of four different schemes: (1) univariate model; (2) model 1, adjusted for age, gender, ICV, and the left Ce; (3) model 2, adjusted for age, gender, ICV, and the left Co; (4) model 3, adjusted for age, gender, ICV, the left Ce and left Co. There was no relevant multicollinearity among these six predictors (all VIF < 5.000).

## 3 Results

### 3.1 Demographics and clinical characteristics

In the current cross-sectional study, a total of 45 patients with CLBP and 45 HCs were included. Demographics and clinical characteristics were presented in Table 2. There was no noteworthy difference between gender, age, and ICV between the two groups.

**Table 2:** Demographic information and clinical data.

	CLBP	HCs	*p*
N	45	45	–
Age	59.0 (52.0 ~ 66.0)	64.0 (53.0 ~ 69.0)	0.332^a^
Gender (male/female)	23/22	30/15	0.134^b^
Pain duration (years)	3 (1 ~ 8)	–	–
VAS	8.1 (7.5 ~ 8.6)	–	–
ODI	64.0 (57.0 ~ 68.0)	–	–
ICV, cm^3^	1484.732 ± 134.848	1523.052 ± 152.762	0.210^c^

Data are represented as mean ± SD or median (quartiles). ^a^The *p*-values were acquired through Mann–Whitney U test. ^b^The *p*-values of the gender distribution were determined with the help of Chi-square test. ^c^The *p*-values were obtained by Student’s *t*-test. CLBP, Chronic low Back Pain; HCs, Healthy controls; VAS, Visual Analogue Scale; ODI, Oswestry Disability Index; ICV, Intracranial Volume.

### 3.2 Amygdala subregion analysis

**Figure 1:**
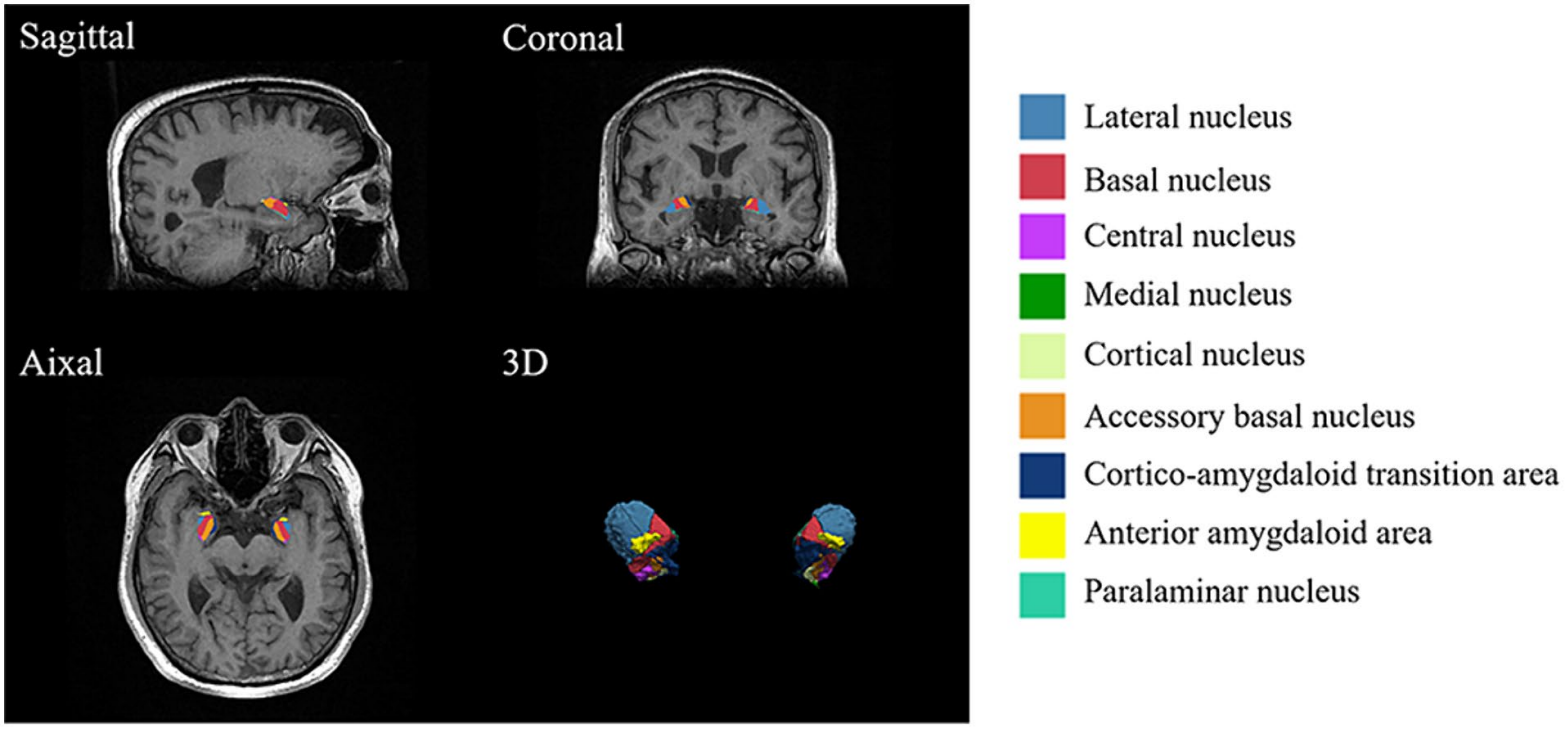
Amygdala subregions. Utilizing FreeSurfer 7.3.2, the amygdala was divided into nine subregions, including the anterior amygdaloid area (AAA), cortico-amygdaloid transition area (CAT), basal nucleus (Ba), lateral nucleus (La), paralaminar nucleus (PL), accessory basal nucleus (AB), medial nucleus (Me), central nucleus (Ce), and cortical nucleus (Co).

The amygdala subregions were shown in Figure 1, and the volume comparison between patients with CLBP and HCs was outlined in Table 3. In comparison to HCs, patients with CLBP displayed a significant increase in the volume of the left Ce and left Co. However, there was no significant difference in the volumes of the whole amygdala, bilateral amygdala, or other amygdala subregions.

**Table 3:** Differences between the whole and individual amygdala volumes of the patients with CLBP and HCs.

	CLBP	HCs	*p*
Whole amygdala, cm^3^	3.244 (3.067 ~ 3.462)	3.242 ± 0.284	0.631^a^
Rt.Whole amygdala, cm^3^	1.633 ± 0.144	1.626 ± 0.152	0.836^b^
Lt.Whole amygdala, cm^3^	1.652 ± 0.153	1.615 ± 0.167	0.282^b^
Lt.La, cm^3^	0.646 ± 0.063	0.640 ± 0.064	0.614^b^
Lt.Ba, cm^3^	0.413 ± 0.044	0.396 (0.376 ~ 0.436)	0.292^a^
Lt.AB, cm^3^	0.242 ± 0.028	0.231 ± 0.026	0.068^b^
Lt.AAA, cm^3^	0.047 ± 0.006	0.045 (0.040 ~ 0.051)	0.614^a^
Lt.Ce, cm^3^	0.043 ± 0.007	0.037 (0.033 ~ 0.044)	*0.002* ^a^
Lt.Me, cm^3^	0.020 ± 0.004	0.018 (0.015 ~ 0.022)	0.062^a^
Lt.Co, cm^3^	0.024 ± 0.004	0.022 ± 0.004	*0.028* ^b^
Lt.CAT, cm^3^	0.168 ± 0.019	0.166 ± 0.019	0.497^b^
Lt.PL, cm^3^	0.049 ± 0.006	0.049 (0.045 ~ 0.053)	0.793^a^
Rt.La, cm^3^	0.631 (0.588 ~ 0.657)	0.640 ± 0.067	0.323^a^
Rt.Ba, cm^3^	0.411 ± 0.040	0.408 ± 0.039	0.738^b^
Rt.AB, cm^3^	0.243 ± 0.026	0.236 ± 0.026	0.177^b^
Rt.AAA, cm^3^	0.048 ± 0.006	0.046 (0.044 ~ 0.051)	0.451^a^
Rt.Ce, cm^3^	0.043 ± 0.006	0.040 ± 0.007	0.069^b^
Rt.Me, cm^3^	0.019 ± 0.005	0.019 (0.016 ~ 0.022)	0.920^a^
Rt.Co, cm^3^	0.024 ± 0.004	0.023 ± 0.003	0.508^b^
Rt.CAT, cm^3^	0.164 ± 0.016	0.164 ± 0.017	0.889^b^
Rt.PL, cm^3^	0.049 ± 0.005	0.049 ± 0.005	0.976^b^

Data are represented as mean ± SD or median (quartiles). ^a^The *p*-values were acquired through Mann–Whitney U test. ^b^The *p*-values were obtained by Student’s *t*-test. CLBP, Chronic low back pain; HCs, Healthy controls; Lt, left; Rt, right; La, Lateral nucleus; Ba, Basal nucleus; AB, Accessory basal nucleus; AAA, Anterior amygdaloid area; Ce, Central nucleus; Me, Medial nucleus; Co, Cortical nucleus; CAT, Cortico-amygdaloid transition area; PL, Paralaminar nucleus. Statistically significant *p*-values are displayed in italics.

### 3.3 Correlation analysis of amygdala subregions with clinical characteristics

Our research did not reveal any significant correlation between the volumes of the left Ce and left Co in patients with CLBP and their clinical scale scores and pain duration (Table 4).

**Table 4:** Correlation analysis of amygdala subregions with clinical characteristics.

	*r*	*p*
Lt.Ce-VAS	0.159	0.296
Lt.Ce-ODI	0.010	0.950
Lt.Ce-pain duration	−0.015	0.924
Lt.Co-VAS	0.132	0.386
Lt.Co-ODI	0.217	0.153
Lt.Co-pain duration	−0.093	0.542

Lt, left; Rt, right; Ce, Central nucleus; Co, Cortical nucleus; VAS, Visual Analogue Scale; ODI, Oswestry Disability Index.

### 3.4 OR risk of amygdala subregions for patients with CLBP

The multivariate logistic regression was employed to assess whether alterations in the volumes of the amygdala subregions were correlated with a higher risk of CLBP. We adjusted for age, gender, ICV, left Ce volume, and left Co volume in the final model (Tables 5, 6). The subjects with an increased volume of the left Ce (OR = 1.095; 95%CI, 1.015–1.181; *p* = 0.02) had a higher risk of being CLBP compared to those with a normal volume of the left Ce.

**Table 5:** Multivariate logistic regression analysis of the association between amygdala subregions and CLBP.

	Model				Variable	
	χ^2^	Degrees of freedom	*p*	Nagelkerke R^2^	OR (95%CI)	*p*
Model 1	13.569	4	*0.009*	0.187		
Age					1.001 (0.946–1.060)	0.969
Gender					0.531 (0.157–1.788)	0.306
ICV					0.092 (0.002–5.411)	0.251
Lt.Ce					1.105 (1.037–1.178)	*0.002*
Model 2	7.704	4	0.103	0.109		
Age					1.009 (0.954–1.068)	0.749
Gender					0.673 (0.206–2.201)	0.513
ICV					0.203 (0.004–9.826)	0.420
Lt.Co					1.147 (1.015–1.297)	*0.028*
Model 3	13.758	5	*0.017*	0.189		
Age					1.004 (0.947–1.065)	0.885
Gender					0.546 (0.160–1.857)	0.332
ICV					0.090 (0.002–5.301)	0.247
Lt.Ce					1.095 (1.015–1.181)	*0.020*
Lt.Co					1.034 (0.890–1.201)	0.664

Model 1, adjusted for age, gender, ICV, and Lt.Ce; Model 2, adjusted for age, gender, ICV, and Lt.Co; Model 3, adjusted for age, gender, ICV, Lt.Ce, and Lt.Co. Lt, Left; Ce, central nucleus; Co, cortical nucleus; ICV, intracranial volume. Statistically significant *p*-values are displayed in italics.

**Table 6:** Univariate logistic regression analysis of the association between amygdala subregions and CLBP.

Variable	OR (95%CI)	*p^#^*	χ^2^	Degrees of freedom	*p* ^*^	Nagelkerke *R*^2^
Age	0.980 (0.934–1.028)	0.407	0.694	1	0.405	0.010
Gender	0.523 (0.223–1.225)	0.136	2.260	1	0.133	0.033
ICV	0.152 (0.008–2.885)	0.210	1.610	1	0.204	0.024
Lt.Ce	1.079 (1.018–1.144)	*0.010*	7.51	1	*0.006*	0.107
Lt.Co	1.135 (1.011–1.273)	*0.032*	4.97	1	*0.026*	0.072

*p*^#^, *p*-value of the variable. p^*^, *p*-value of the model. Lt, Left; Ce, central nucleus; Co, cortical nucleus; ICV, intracranial volume. Statistically significant *p*-values are displayed in italics.

## 4 Discussion

As far as we know, this is the first study to analyze structural alterations of the subregions of the amygdala in patients with CLBP. In comparison to the HCs, patients with CLBP had an increase in volume in the left Ce and left Co. Moreover, an increased volume of the left Ce predisposed individuals to a higher risk of CLBP.

In this study, patients with CLBP were found to have an increased volume of the left Ce. Moreover, the increase in the left Ce raises the risk of being CLBP. The Ce of the amygdala (CeA), a nociceptive center in the brain, receives nociceptive information from two main pathways, the first of which originates in the basolateral amygdala (BLA) (30, 37). The central nervous system transmits noxious information resulting from past painful experiences and mood states from the BLA to the CeA (BLA-CeA pathway) (38). By activating the BLA-CeA pathway, reward-related behavior is enabled, thus resulting in pain inhibition (39). Meanwhile, an overstimulation of glutamate (Glu) neurons in the BLA due to noxious stimuli can lead to an excitation of Glu neurons in the ventral hippocampus (vHIP), thus activating GABA neurons in the CeA via the vHIP-CeA pathway (38, 40), which may be a significant factor in Chronic pain. Meanwhile, the second pathway transmits more direct and raw nociceptive information to the CeA via the laterocapsular region of the CeA (CeLC) (41). The CeLC receives pain signals transmitted via the spino-parabrachio-amygdaloid pathway, and may also via direct projections from the spinal cord (42–44). It is noteworthy that the parabrachial input has peptidergic features and is the exclusive source of calcitonin gene-related peptide (CGRP) in the amygdala. Moreover, the CeA is the main output nucleus of the amygdala. GABAergic projection neurons are present in the CeLC, which also contain peptide substances like corticotropin-releasing factor (CRF) (41). Neurons in the CeA, which contain CRF, are innervated by terminals that contain CGRP from the parabrachial region (45–47) and project to distinct regions in the basal forebrain and brainstem (48). It is thought that an abnormal activation of these neurons in the CeA is a main contributor to chronic pain (49). The involvement of the Ce in the pathophysiological mechanism of CLBP is evident from these findings.

Interestingly, our study also observed increased volume of the left Co in patients with CLBP. However, the relationship between Co. and pain is not well understood at present. Co, located in the superficial region of the amygdala, is directly connected to the olfactory system and is involved in the processing of olfactory stimuli (50, 51). Nociception and olfaction are thought to intersect at the sensory, behavioral and emotional levels, as well as the molecular level (52). This is evidenced by the co-expression of pain genes in olfactory brain structures, the interaction of ion channels or G protein-coupled receptors in the transmission and processing of pain and olfaction, and the shared brain regions involved in central processing of nociceptive and olfactory information (52). Meanwhile, previous research has stressed the effect of odors on the qualitative evaluation of pain (53–56). Therefore, the abnormal volume changes in the left Co may impact CLBP by influencing olfaction. Thus, it is essential to examine the changes in olfaction in patients with CLBP and their association with CLBP in future studies.

It should be noted that Ce and Co were both situated in the left amygdala. Unlike the right amygdala, which is thought to be linked to pain promotion, the left amygdala does not usually have a pain-promoting capacity, and may even be associated with pain suppression (57). In the future, it is essential to investigate whether the lateralization of the subregions of the amygdala in this study promotes or inhibits the production of CLBP.

With the exception of the left Ce and left Co, we did not detect any significant variations in the volumes of other amygdala subregions between the groups. Our research also did not demonstrate a relationship between the volume of the left Ce and left Co and clinical scale scores and pain duration. This suggested that these two amygdala subregions could be a phenotypic biomarker of CLBP and may play a role in the pathophysiology of CLBP. However, it also raises the possibility that the abnormal volume changes in these amygdala subregions could be attributed to other factors, such as analgesic medication. The Ce is thought to be involved in tolerance to the antinociceptive effect of NSAIDs (58). Meanwhile, it is believed that Co plays a part in post-stress analgesia (59). Consequently, more research is needed in the future to rule out the influence of analgesics on the volume of the amygdala subregions.

This research has several limitations. To begin with, since this was a cross-sectional study, it is not possible to establish a causal relationship between changes in the amygdala subregions and CLBP. Therefore, further longitudinal research is necessary. Additionally, this did not allow us to examine the effect of medication on the changes of the amygdala and its subregions in CLBP. Finally, this study only concentrated on the structural changes of the amygdala subregions in patients with CLBP, not on the functional changes. Consequently, it is essential to investigate the functional changes of the amygdala subregions in patients with CLBP in the future.

## 5 Conclusion

In conclusion, our research has, for the first time, demonstrated that altered volumes of the left Ce and left Co in the amygdala subregions may be involved in the pathogenesis of CLBP. Furthermore, the volume of the left Ce may serve as a tool for identifying patients with CLBP and to at risk to develop CLBP.

## Data availability statement

The original contributions presented in the study are included in the article/supplementary material, further inquiries can be directed to the corresponding authors.

## Ethics statement

The studies involving humans were approved by the Yancheng Third People’s Hospital Ethics Committee. The studies were conducted in accordance with the local legislation and institutional requirements. The participants provided their written informed consent to participate in this study. Written informed consent was obtained from the individual(s) for the publication of any potentially identifiable images or data included in this article.

## Author contributions

S-YG: Data curation, Writing – original draft. F-CS: Data curation, Writing – original draft. SW: Data curation, Writing – original draft. C-YW: Data curation, Writing – review & editing. X-XY: Data curation, Writing – review & editing. Y-FS: Data curation, Writing – review & editing. J-BH: Data curation, Writing – review & editing. FC: Conceptualization, Methodology, Supervision, Writing – review & editing. P-LP: Conceptualization, Methodology, Supervision, Writing – review & editing. W-HL: Conceptualization, Methodology, Supervision, Writing – review & editing.

## Footnotes

^1^ https://surfer.nmr.mgh.harvard.edu/
